# Paranasal Sinus Morphometry for Forensic Sex Estimation: A Computed Tomography Study of 499 Individuals with a Cross-Validated, Transparently Reported Machine Learning Model

**DOI:** 10.3390/diagnostics16121928

**Published:** 2026-06-22

**Authors:** Muhammet Can, Cihangir Işık, Burcu Düzel Asıg

**Affiliations:** 1 Department of Forensic Medicine, Faculty of Medicine, Balikesir University, 10145 Balikesir, Türkiye; 2 Forensic Medicine Division, Balıkesir Atatürk City Hospital, 10100 Balıkesir, Türkiye; 3 Department of Radiology, Defne Hospital, 31000 Hatay, Türkiye; drburcuduzel@gmail.com

**Keywords:** forensic anthropology, logistic regression, machine learning, paranasal sinuses, sex determination, sex estimation, computed tomography

## Abstract

**Background/Objectives:** Paranasal sinus morphometry on computed tomography (CT) is of interest for forensic sex estimation, but many published predictive models rely on in-sample formulas without cross-validation, external testing, or release of model parameters. We aimed to characterize sex differences, pneumatization patterns, asymmetry, and age relationships of the paranasal sinuses in a Turkish adult population, and to develop, cross-validate, and transparently report a predictive model for sex estimation, explicitly benchmarked against the single best morphometric feature. **Methods:** In this single-center, STROBE-compliant retrospective cross-sectional study, maxillary, frontal, and sphenoid sinus volumes were measured by semi-automated active-contour segmentation in ITK-SNAP on CT scans of 499 adults (282 male, 217 female; 18–65 years). Between-sex differences were tested with the Mann–Whitney U test with Bonferroni correction; effect sizes used Cliff’s delta and the probability of superiority. L1-regularized logistic regression, random forest, and gradient boosting were trained with 10-fold stratified cross-validation and a held-out 20% test set, and compared with a univariate frontal-volume benchmark. **Results:** All three sinus volumes were larger in males (all Bonferroni-adjusted *p* < 0.001), with the largest effect among the individual sinuses for the frontal sinus (Cliff’s delta = 0.53; probability of male superiority = 0.77). The best classifier was L1-regularized logistic regression (10-fold cross-validated AUC 0.79 ± 0.07; held-out test AUC 0.80; accuracy 70%). Because the area under the ROC curve of a single continuous marker equals its probability of superiority, frontal volume alone reached an AUC of approximately 0.77; the multivariable model therefore added little beyond this single feature. Age could not be reliably estimated (test mean absolute error ≈ 10.8 years; R^2^ ≈ 0). **Conclusions:** Paranasal sinus volumes show robust sex dimorphism, concentrated in the frontal sinus, but provide only moderate sex discrimination—appropriate as one corroborating input in a forensic identification workflow rather than a stand-alone determinant. Age cannot be reliably estimated from sinus morphometry in this cohort. Full model coefficients are reported to permit independent replication.

## 1. Introduction

The paranasal sinuses show marked inter-individual variability in volume, pneumatization pattern, and asymmetry that has long interested forensic anthropologists [1]. Because they are protected within bone, the sinuses can survive trauma, fire, and decomposition that destroy soft-tissue features, which makes them attractive structures for sex estimation and individual identification in degraded remains [2,3]. Computed tomography (CT) now permits accurate, reproducible volumetric measurement of these structures and has largely replaced two-dimensional radiographic indices.

The biological basis of these differences lies in the development and maturation of the sinuses. The paranasal sinuses are rudimentary at birth and enlarge progressively through childhood and adolescence by pneumatization of the surrounding bone, essentially reaching their adult size once skeletal growth is complete around 18–20 years of age [4,5]. After maturity, the sinuses are relatively stable, although gradual bone remodeling, dental loss, and aging of the maxilla can produce modest, mainly later-life changes in maxillary volume [5]. Sexual dimorphism in sinus volume is generally attributed to the larger overall craniofacial dimensions of males and to sex differences in growth duration and bone remodeling, with the frontal sinus typically showing the most pronounced male–female difference [6,7]. Sinus morphology and volume are additionally influenced by factors such as population ancestry, nasal and sinus pathology, prior surgery, and individual anatomical variation in pneumatization. Because pneumatization is still incomplete in children and adolescents, and because these maturational and pathological factors would otherwise confound volumetric comparisons, the present study was restricted to adults aged 18–65 years without sinus pathology, so that the observed differences can be interpreted primarily in terms of sex.

Beyond their biological basis, the paranasal sinuses are of particular forensic-anthropological interest: their high inter-individual variability and their resistance to fire, trauma, and decomposition make them valuable not only for sex estimation but also for positive identification through frontal-sinus pattern matching, particularly when the pelvis and cranial vault are damaged or absent. This anthropological rationale, and not methodological critique alone, motivates the continued refinement of sinus-based methods. Despite the substantial literature, the predictive modeling underlying sinus-based sex estimation has often been methodologically limited. Many studies report sex-classification accuracies derived from formulas fitted and evaluated on the same sample, without cross-validation or a held-out test set, and few release their model parameters so that others can reproduce or externally validate the model [8]. Reported accuracies in the 80–90% range may therefore be optimistic relative to performance achievable in independent data. Modern reporting guidance for clinical and forensic prediction models emphasizes cross-validation, separation of training and testing data, calibration assessment, and transparent release of model coefficients [8,9].

In this study, we characterize sex dimorphism, pneumatization patterns, asymmetry, and age relationships of the paranasal sinuses in a Turkish adult cohort, and we develop, cross-validate, and transparently report a predictive model for sex estimation. We deliberately benchmark the multivariable model against the single most discriminative morphometric feature, so that the incremental value of the full model is made explicit. All trained coefficients are released to permit independent replication.

## 2. Materials and Methods

### 2.1. Study Design and Setting

This single-center retrospective cross-sectional study was conducted at the Forensic Medicine Division of Balıkesir Atatürk City Hospital, Türkiye, and is reported in accordance with the STROBE statement for observational studies [10]; aspects relating to model development and reporting follow the TRIPOD recommendations [9]. The protocol was approved by the Hatay Mustafa Kemal University Non-Interventional Clinical Research Ethics Committee (decision 39, meeting 06, 30 April 2025) and conducted in accordance with the Declaration of Helsinki. Because only anonymized existing CT scans were analyzed, the committee waived the requirement for informed consent. The imaging studies analyzed in this work were performed between January 2022 and December 2024.

### 2.2. Participants

We screened all consecutive head CT scans performed in adults for non-paranasal indications such as headache, head trauma without facial fracture, or evaluation of vertigo. Inclusion criteria were age of 18–65 years and absence of sinus pathology on imaging. The lower age bound was chosen because skeletal maturation of the paranasal sinuses is essentially complete by 18 years [4], and the upper bound limits the influence of late-life bone remodeling on maxillary volume [5]. Exclusion criteria were prior sinus surgery, facial trauma, congenital craniofacial anomaly, sinus opacification suggesting acute or chronic sinusitis, and motion artifact precluding accurate segmentation. After applying these criteria, 500 scans remained; 1 case with missing sex information in the imaging record was excluded, yielding a final sample of 499 (282 male, 217 female).

### 2.3. Sample Size

An a priori sample-size calculation was performed for the primary comparison (between-sex difference in maxillary sinus volume). With an anticipated standardized effect size of 0.5 [11], a two-sided alpha of 0.0125 (Bonferroni correction across four primary volumetric outcomes) and 90% power, a Mann–Whitney U test requires approximately 100 individuals per group. A larger sample was deliberately enrolled to enable cross-validated predictive analyses, following the recommendation of at least 50 observations per predictor per outcome class [12]. In addition to this comparison-based calculation, the adequacy of the sample for prediction-model development was assessed directly. With ten candidate predictor parameters (three sinus volumes and the one-hot indicators for pneumatization pattern and asymmetry) and 217 events in the minority (female) class, the data provided approximately 21.7 events per candidate parameter, comfortably exceeding the minimum of ten events per parameter widely recommended for stable multivariable model development and consistent with the formal sample-size criteria of Riley et al. [12]. The recruited cohort therefore provided ample power for both descriptive and predictive analyses.

### 2.4. CT Image Acquisition

All scans were performed on a single multidetector CT scanner (Siemens SOMATOM Sensation 64; Siemens Healthineers, Erlangen, Germany) with a standardized protocol: 120 kV, 100 mAs, 0.6 mm slice thickness, 0.4 mm reconstruction interval, and bone reconstruction kernel. Images were exported in Digital Imaging and Communications in Medicine (DICOM) format.

### 2.5. Sinus Segmentation and Volume Measurement

**Figure 1:**
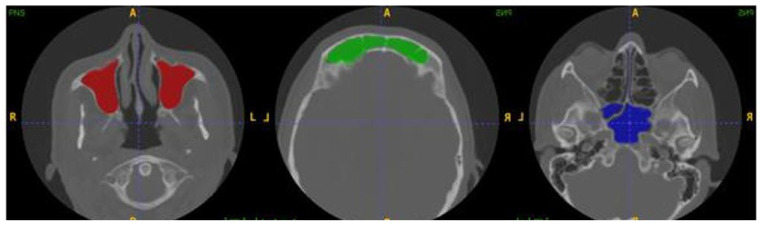
Representative axial computed tomography slices illustrating semi-automated active-contour segmentation of the maxillary (red), frontal (green), and sphenoid (blue) sinuses in ITK-SNAP. The letters indicate anatomical orientation (A = anterior, R = right, L = left, PNS = posterior nasal spine), and the dotted lines represent the orthogonal reference (crosshair) lines of the CT viewer used during segmentation. The colored areas (red, green, and blue) indicate the segmented regions of interest.

Maxillary, frontal, and sphenoid sinuses were segmented using ITK-SNAP version 3.8.0 [13] via its semi-automated active-contour (snake) tool with manual correction of overflow into adjacent structures (Figure 1). ITK-SNAP was selected because its semi-automated active-contour algorithm is well established and validated for paranasal sinus volumetry, permits slice-by-slice manual verification, and does not require the large annotated training datasets that supervised deep learning segmentation would demand; fully automated Python-based and deep learning segmentation pipelines were considered but were not adopted for the present study and are identified as a direction for future work. The inferior boundary of the frontal sinus was defined at the level of the frontal recess, taken as the most caudal axial slice on which an air-containing cavity continuous with the frontal sinus remained enclosed by frontal bone; the nasofrontal duct communication and the ethmoidal air cells were excluded from the segmented volume. To prevent leakage of the active contour into adjacent air-containing spaces, intensity limits corresponding to the air-density range were applied, every segmentation was inspected in all three orthogonal planes (axial, coronal, and sagittal), and any voxels extending beyond the bony sinus walls were manually corrected; all frontal-sinus segmentations were additionally checked against the bony margins to confirm that no overflow into the ethmoid air cells or nasal cavity had occurred. To make the workflow reproducible, the following protocol was applied identically to every scan. Within the ITK-SNAP active-contour module, the pre-segmentation intensity threshold was set to an air-density window of −1024 to −400 Hounsfield units, which retained the air-filled sinus lumen while excluding mucosa and bone. One or more spherical seed bubbles were then placed manually inside each sinus air space, and the region-competition snake was evolved until it filled the cavity and stabilized against the bony walls, evolution being stopped when no further expansion occurred. Leakage was operationally defined as extension of the evolving contour beyond a visible bony wall into an adjacent air-containing space (ethmoid cells or nasal cavity) and was corrected manually on a slice-by-slice basis in all three orthogonal planes. Segmentations were performed by the first author, a forensic medicine specialist with three years of experience in cranial CT morphometry. To assess reliability, 50 randomly selected scans (10% of the cohort) were independently re-segmented by the second author, a board-certified radiologist, blinded to the primary measurements. Inter-observer agreement was quantified using the intraclass correlation coefficient (ICC; two-way mixed-effects model, absolute agreement, single rater) and the Dice similarity coefficient for spatial overlap. Intra-observer reliability was additionally assessed: the first author re-segmented the same 50 scans after an interval of approximately two weeks, blinded to the initial measurements, and intra-observer agreement was quantified with the same ICC model. Intra-observer agreement was excellent for all three sinuses (ICC = 0.998 for maxillary, 0.996 for frontal, and 0.999 for sphenoid volume). The Dice similarity coefficient (DSC = 2|A∩B|/(|A| + |B|), where A and B denote the two observers’ voxel sets for a given sinus) was computed in Python directly from the exported binary segmentation masks, using SimpleITK to read and align the label maps and NumPy to perform the voxel-wise overlap calculation across all 50 paired segmentations. Volumes were recorded in mm^3^ and are reported in cm^3^ throughout (1 cm^3^ = 1000 mm^3^).

### 2.6. Pneumatization and Asymmetry Classification

**Figure 2:**
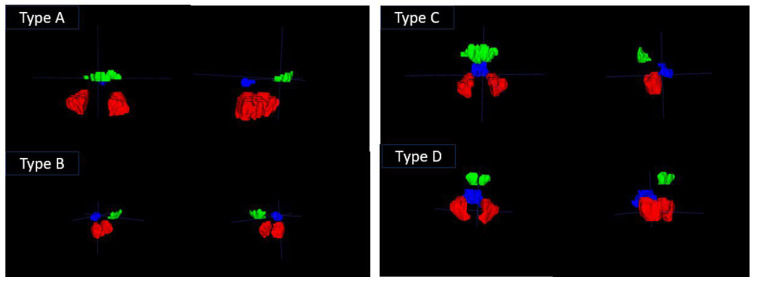
Schematic three-dimensional reconstructions of sphenoid sinus pneumatization patterns according to the classification of Hammer and Radberg: Type A (conchal), Type B (presellar), Type C (incomplete sellar), and Type D (complete sellar).

Sphenoid sinus pneumatization was classified according to the system of Hammer and Radberg [14] as Type A (conchal), Type B (presellar), Type C (incomplete sellar), or Type D (complete sellar) (Figure 2). Maxillary asymmetry was categorized as left-dominant, right-dominant, or symmetric based on the larger side. Asymmetry was assessed metrically from the segmented volumes rather than visually. For each individual, an asymmetry index was computed as the absolute difference between the right and left maxillary sinus volumes divided by the mean of the two sides and expressed as a percentage. Individuals whose index was 10% or less were classified as symmetric; the remaining individuals were assigned to the side with the larger volume (right- or left-dominant).

### 2.7. Statistical Analysis

Distributions were assessed for normality using the Shapiro–Wilk test stratified by sex. Because every sinus volume was significantly non-normal and the Levene test demonstrated unequal variances between sexes, between-sex differences were tested with the Mann–Whitney U test; Welch’s *t*-test results are also reported for comparability with the prior literature. Effect sizes were quantified using Cliff’s delta and the probability of male-superior values [15]. The Bonferroni method corrected for multiple testing across the four primary volumetric outcomes (maxillary, frontal, sphenoid, total). Differences across pneumatization patterns were tested with the Kruskal–Wallis test, correlations with Spearman’s rho, and categorical associations with Pearson’s chi-square test. All tests were two-sided with alpha 0.05. Analyses were performed in Python 3.11 (Python Software Foundation, Wilmington, DE, USA) using SciPy 1.14 and scikit-learn 1.5. All computations were run under the Anaconda distribution on a 64-bit Windows 11 workstation and additionally used NumPy 1.26, pandas 2.2, and statsmodels 0.14; SimpleITK 2.3 was used for handling the segmentation label maps and computing Dice coefficients.

### 2.8. Predictive Modeling for Sex and Univariate Benchmark

Features comprised the three sinus volumes plus one-hot encodings of pneumatization pattern (A/B/C/D) and asymmetry (left/none/right), giving 10 input variables. Numerical features were z-standardized using training-set parameters only. Three classifier families were compared: L1-regularized logistic regression, random forest, and gradient boosting. The dataset was first partitioned into an 80%/20% stratified train/test split, with the test set held out from all training and hyperparameter selection. On the training partition, 10-fold stratified cross-validation with grid search selected hyperparameters (regularization strength C from {0.01, 0.1, 1, 10} and penalty from {L1, L2} for logistic regression; n_estimators from {100, 300} and max_depth from {none, 5, 10} for random forest; n_estimators from {100, 200}, max_depth from {2, 3, 4}, and learning_rate from {0.05, 0.1} for gradient boosting). The selected model was retrained on the full training partition and evaluated once on the held-out test set. Hyperparameter optimization was performed with scikit-learn’s GridSearchCV, an exhaustive grid search over the ranges listed above, scored by ROC-AUC within the 10-fold stratified cross-validation; no separate optimization framework such as Optuna, HyperOpt, or Bayesian search was used. The 80/20 split was chosen so that the held-out test set (*n* = 100) remained large enough for stable estimation of discrimination metrics while preserving the maximum number of cases for model training; reliance on any single split was further reduced because all model selection was carried out inside the 10-fold cross-validation rather than on the held-out partition. Both the train/test partition and the cross-validation folds were stratified by sex to preserve the male-to-female ratio across all partitions. Age was not used as a stratification factor because it was not an outcome of the sex-prediction model; however, the male and female subgroups had closely comparable age distributions (Section 3.1), which limits confounding of the sex effect by age. Discrimination was reported as the area under the receiver operating characteristic curve (AUC); calibration was reported as the Brier score and a reliability plot using out-of-fold predictions [16]. Feature importance was extracted from the random forest classifier for interpretability.

To quantify the incremental value of the multivariable model, we defined a univariate benchmark using the single most discriminative feature (frontal volume). For a single continuous marker, the AUC is mathematically equal to the probability of superiority (the common-language effect size); we therefore report the frontal-only AUC directly from this quantity and compare it with the full model. The final logistic regression model was refit on all 499 cases, and its full coefficient set is reported in Supplementary Table S1, and the procedure for applying the model is given in Supplementary Section S1, to permit independent replication.

### 2.9. Exploratory Age-Estimation Model

As a secondary, exploratory analysis, the same feature set was used as input to linear regression, random forest regression, and gradient-boosting regression for age. Performance was reported as mean absolute error (MAE), root mean squared error (RMSE), and coefficient of determination (R^2^) on the held-out test set and as 10-fold cross-validated MAE on the full dataset.

## 3. Results

### 3.1. Sample Characteristics

The 499 included individuals had a median age of 33 years (interquartile range [IQR] 26–44; range 18–65). Of these, 282 (56.5%) were male and 217 (43.5%) female. Male and female participants had closely comparable age distributions (male 35.7 ± 12.2 years; female 34.9 ± 12.2 years; Mann–Whitney U test *p* = 0.39), indicating that the between-sex differences in sinus volume reported below are not confounded by a difference in age between the groups. Pneumatization patterns were Type D in 292 (58.5%), Type C in 168 (33.7%), Type B in 36 (7.2%), and Type A in 3 (0.6%). Asymmetry was left-dominant in 201 (40.3%), right-dominant in 183 (36.7%), and symmetric in 115 (23.0%). Detailed characteristics are presented in Table 1.

### 3.2. Inter-Observer Reliability

Across 50 scans independently re-segmented by the second observer, the ICC (two-way mixed, absolute agreement) for total sinus volume was 0.96 (95% confidence interval [CI] 0.94–0.98), indicating excellent agreement. ICCs for individual sinuses were 0.94 (maxillary), 0.91 (frontal), and 0.93 (sphenoid). The mean Dice similarity coefficient for spatial overlap was 0.91 ± 0.03.

### 3.3. Distributional Properties

Shapiro–Wilk tests rejected normality for all three sinus volumes within both sex strata (all *p* ≤ 0.05; frontal volume showed the strongest right-skew with *p* < 0.001). Levene’s tests demonstrated unequal variances between sexes for every volume (all *p* < 0.05). Non-parametric tests were therefore used for all between-group comparisons.

### 3.4. Sex Differences in Sinus Volume

**Figure 3:**
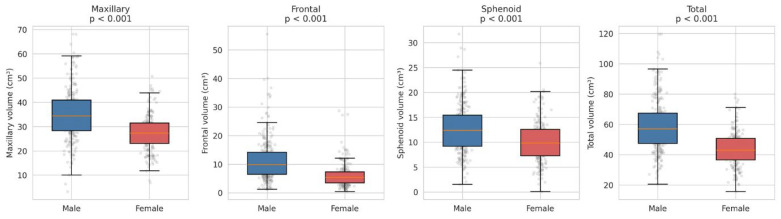
Distribution of maxillary, frontal, sphenoid, and total sinus volumes by sex, shown as box plots with overlaid raw observations. All four comparisons are significant at Bonferroni-adjusted *p* < 0.001 by Mann–Whitney U.

All three sinus volumes were significantly larger in males than in females (Table 2; all Bonferroni-adjusted *p* < 0.001). Among the individual sinuses, the largest standardized effect was for the frontal sinus (Cliff’s delta = 0.53; probability of male superiority = 0.77), followed by maxillary (delta = 0.45) and sphenoid (delta = 0.33); the composite total volume showed the largest effect overall (delta = 0.56). Welch’s *t*-test produced concordant inferences (Figure 3).

### 3.5. Pneumatization, Asymmetry, and Age

**Figure 4:**
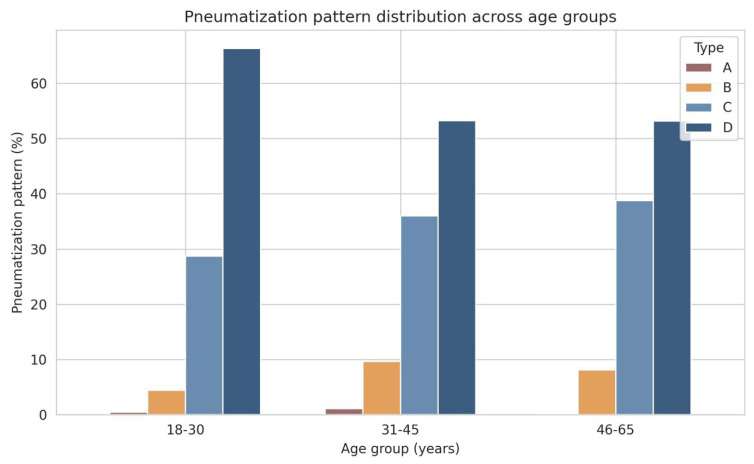
Distribution of pneumatization patterns within age groups (18–30, 31–45, 46–65 years). The descriptive trend toward fewer Type D individuals in older groups did not reach statistical significance (chi-square, *p* = 0.077).

Kruskal–Wallis tests demonstrated strong differences in volume across pneumatization patterns, most pronounced for the sphenoid sinus (H = 241, *p* < 0.001), where median sphenoid volume rose monotonically from Type A (1.5 cm^3^) to Type B (5.1 cm^3^) to Type C (8.5 cm^3^) to Type D (13.3 cm^3^). Maxillary (H = 32, *p* < 0.001) and frontal (H = 24, *p* < 0.001) volumes were also greater in higher pneumatization grades but with smaller magnitudes. Although Type D was the most common pattern in every age group, the association between pneumatization pattern and age group did not reach significance (chi-square = 11.4, df = 6, *p* = 0.077; Figure 4), nor did the association between pneumatization and sex (chi-square = 4.9, df = 3, *p* = 0.18). Left-dominance was slightly more frequent than right-dominance overall (40.3% vs. 36.7%), but the difference was not significant between sexes (chi-square = 4.4, df = 2, *p* = 0.11). Spearman’s correlations between age and sinus volumes were weak: maxillary rho = −0.13 (*p* = 0.005), frontal rho = +0.06 (*p* = 0.21), and sphenoid rho = −0.05 (*p* = 0.23). The statistically significant maxillary correlation corresponds to an R^2^ of approximately 0.016, so age explains less than 2% of the variance in maxillary volume.

### 3.6. Inter-Sinus Correlations

Sinus volumes were moderately and positively intercorrelated: maxillary–frontal rho = 0.41, maxillary–sphenoid rho = 0.39, and frontal–sphenoid rho = 0.41 (all *p* < 0.001).

### 3.7. Predictive Model Performance for Sex Estimation

**Figure 5:**
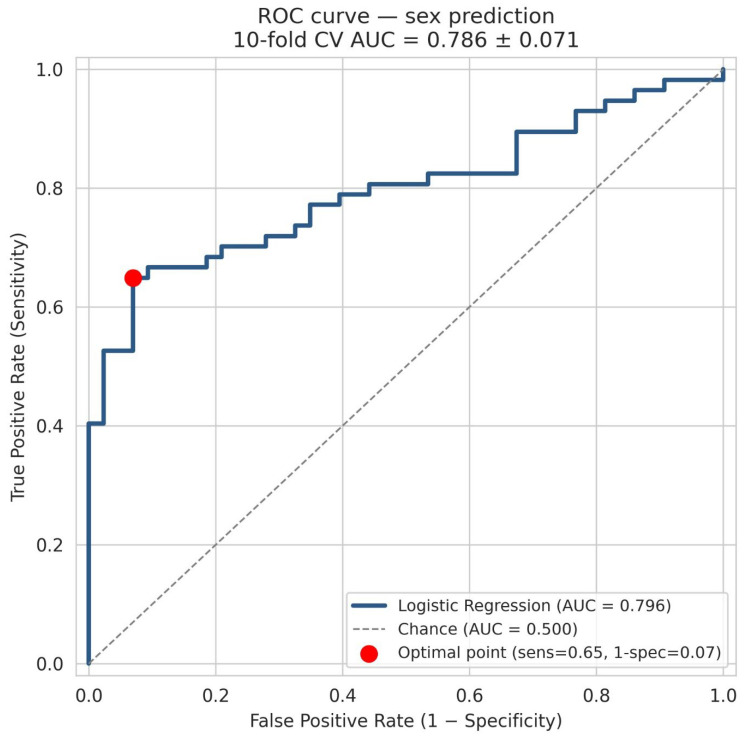
Receiver operating characteristic curve of the logistic regression sex-prediction model on the held-out test set (*n* = 100). The marker indicates the Youden J optimal operating point; the dashed line represents chance performance.

**Figure 6:**
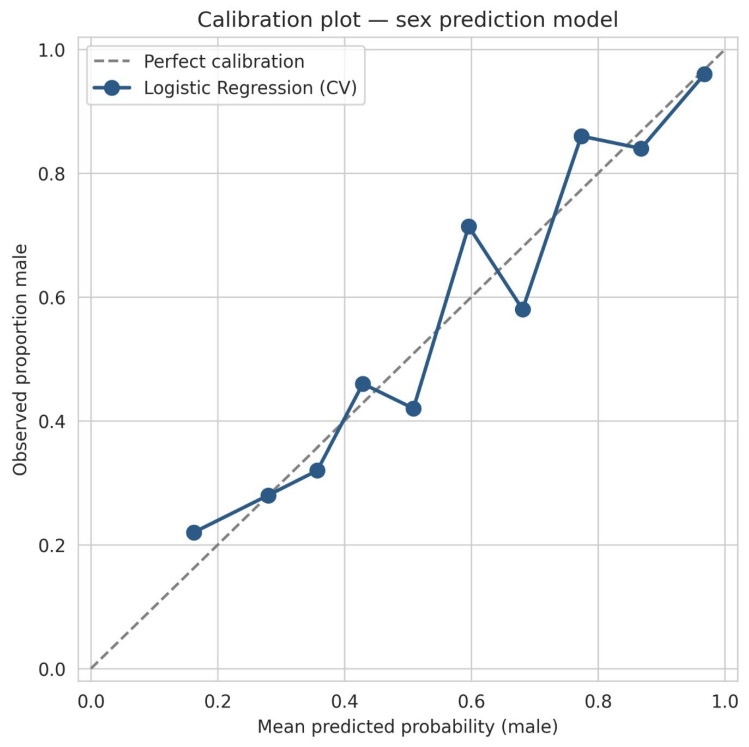
Calibration plot for the logistic regression model using out-of-fold predictions across 10-fold cross-validation. The dashed line indicates perfect calibration; predicted probabilities deviate only mildly from the diagonal.

Of the three classifier families evaluated by 10-fold cross-validation on the training partition, L1-regularized logistic regression achieved the highest mean cross-validated AUC (0.789), marginally above random forest (0.782) and gradient boosting (0.775). On the held-out 20% test set, the logistic regression model obtained AUC = 0.796, accuracy 70%, sensitivity 0.74, specificity 0.65, precision 0.74, and F1 score 0.74 (Figure 5, Table 3). The Brier score was 0.18, indicating modest calibration; the reliability plot (Figure 6) showed predicted probabilities close to the diagonal across the full range. Random forest feature importance ranked frontal sinus volume highest (0.39), followed by maxillary (0.32) and sphenoid (0.20); pneumatization and asymmetry variables together contributed less than 10% of total importance. The model-explainability analysis conveys a consistent and interpretable message: the three continuous sinus volumes essentially carried all of the discriminative signal, with the frontal sinus contributing the most and the categorical pneumatization and asymmetry features contributing negligibly. The dominance of frontal volume mirrors its position as the feature with the largest univariate sex effect (Cliff’s delta = 0.53), so the learned model is, in effect, reweighting a single strongly dimorphic measurement rather than relying on a complex or opaque combination of variables. This concordance between the univariate effect sizes and the multivariable feature ranking supports the biological plausibility of the model and indicates that its decisions can be traced transparently to an anatomically meaningful, sexually dimorphic volume difference rather than to spurious feature interactions.

The univariate benchmark clarified the source of this performance. Because the AUC of a single continuous marker equals its probability of superiority, frontal volume alone yields an AUC of approximately 0.77. The full 10-variable model improved discrimination only marginally beyond this value (cross-validated AUC 0.79; test AUC 0.80), indicating that most of the discriminative information resides in a single feature and that the additional variables and the choice of a more flexible learner contribute little incremental value.

### 3.8. Exploratory Age Estimation

Age regression performed near chance across all classifier families. The best held-out test MAE was 10.8 years (random forest), with cross-validated MAE of 10.2 years and R^2^ values consistently near zero or negative. The available features therefore do not support reliable age estimation in this cohort.

## 4. Discussion

This study analyzed CT-derived paranasal sinus morphometry in 499 adults from a Turkish population and developed a cross-validated predictive model for sex estimation that was explicitly benchmarked against the single best feature. Three findings deserve emphasis.

First, robust sex dimorphism is concentrated in the frontal sinus. All three sinuses were larger in males, but among the individual sinuses the largest non-parametric effect size was for the frontal sinus (Cliff’s delta = 0.53). This concurs with prior reports identifying the frontal sinus as a reliable structure for sex estimation [6,7] and supports its use as the primary discriminative feature. The smaller sphenoid effect (delta = 0.33) and the strong dependence of sphenoid volume on pneumatization pattern (Kruskal–Wallis H = 241) together suggest that sphenoid volume is modulated more by individual anatomical variation than by sex. It is worth noting that, although the frontal sinus produced the largest sex effect, its median volume (7.1 cm^3^) was lower than that of the sphenoid sinus (11.1 cm^3^). This is consistent with the well-recognized status of the frontal sinus as the most anatomically variable of the paranasal sinuses—frequently hypoplastic and occasionally aplastic—so that its central tendency is lower than that of the more consistently pneumatized sphenoid sinus, even though the frontal sinus discriminates the sexes more strongly. The finding therefore reflects normal anatomical variation in the frontal sinus rather than a measurement or segmentation artifact, and a comparable ordering of mean volumes has been reported in other CT-based volumetric series.

Second, the multivariable model adds little beyond a single feature, and pneumatization and asymmetry are descriptive rather than predictive. The univariate benchmark showed that frontal volume alone reaches an AUC of approximately 0.77, while the full 10-variable model reached only 0.79–0.80. Although Type D pneumatization is descriptively more common in younger adults and is associated with larger total sinus volumes, chi-square tests did not support significant associations between pneumatization and either sex (*p* = 0.18) or age group (*p* = 0.077), and the combined contribution of pneumatization and asymmetry variables to feature importance was below 10%. A parsimonious model based on frontal volume is therefore preferable on grounds of interpretability and transferability, and the categorical features should not be over-interpreted as independent forensic markers, as some previous work has implied [17].

Third, age cannot be reliably estimated from sinus morphometry alone. Despite a statistically significant correlation between age and maxillary volume (rho = −0.13, *p* = 0.005), the corresponding R^2^ is below 2%, and our trained regressors achieved MAE near 10 years with R^2^ close to zero on cross-validation. Earlier reports of accurate age estimates from sinus volumes [18] may partly reflect optimistic bias arising from in-sample evaluation. We report this negative finding deliberately, because publication of negative results is important to prevent overconfidence in forensic age-estimation methods [19]. The same evidence explains why age was not included as a predictor in the sex-classification model: age was only weakly correlated with sinus volume (all |rho| ≤ 0.13, explaining under 2% of the variance), the male and female groups had comparable age distributions (Section 3.1), and sinus volumes changed little across the adult age range in either sex. Adding age as a feature was therefore unlikely to improve sex discrimination and would have introduced a variable that is itself frequently unknown in forensic casework; sex estimation was consequently based on the sinus measurements alone.

Comparison with previous predictive models is instructive. Earlier studies have reported sex-classification accuracies of 80–90% from sinus morphometry [20,21,22], but most lacked train/test separation and cross-validation. Our held-out test accuracy of 70% and cross-validated AUC of 0.79 are more conservative and are consistent with the inherent overlap between male and female sinus-volume distributions: even for the most discriminative single feature, the probability of male superiority is 0.77, meaning that approximately 23% of male–female pairs cannot be correctly ordered by frontal volume alone. Performance estimated without held-out evaluation tends to be optimistic, and the present figures are likely closer to what is achievable in independent data.

These results carry direct forensic implications. An AUC of 0.79 corresponds to moderate discrimination. In a forensic identification workflow, this places sinus morphometry as one corroborating input among several—such as cranial metric and non-metric traits, pelvic morphology, and DNA analysis when available—rather than as a stand-alone determinant. We emphasize that this level of performance is appreciably lower than the sex-classification accuracies typically achieved with established pelvic indicators (commonly above 90%) [23] and cranial indicators (commonly in the 80–90% range) [24,25], and we do not propose that sinus morphometry should be regarded as equivalent in utility to these skeletal regions. Its principal value is instead as a supplementary indicator in situations where the pelvis and cranial vault are fragmentary, burned, or otherwise unavailable, whereas the sinuses—protected within surrounding bone—may remain intact and measurable. Sinus morphometry should accordingly be interpreted as complementary to, not a replacement for, the standard pelvic and cranial methods. In keeping with current reporting recommendations, we describe the final classifier specifically as a regularized logistic regression model rather than under the broader label of artificial intelligence, because the distinction between learned models and hand-tuned formulas has not always been explicit in the literature [9]. We also report effect sizes and calibration alongside *p* values, and we release the trained model coefficients (Supplementary Table S1) so that the community can attempt replication.

These results should also be viewed against the recent move toward deep learning approaches to craniofacial sex estimation. Convolutional neural networks applied directly to cranial CT data have reported markedly higher accuracies than volumetric models such as ours; for example, a deep learning framework evaluated on three-dimensional cranial CT scans reached 97% classification accuracy and outperformed a human assessor [26], and a deep feature-selection and feature-fusion model applied to skull CT images reported accuracies above 96% [27]. Such models, however, typically learn from the full three-dimensional shape and texture of the cranium rather than from a small set of interpretable volumes, require large training datasets and substantial computation, and often function as opaque, less reproducible predictors whose decisions are difficult to audit. Our study deliberately occupies the opposite end of this trade-off: it uses three transparent, anatomically meaningful measurements and a fully reported linear model whose coefficients are released for independent replication. We therefore do not present sinus volumetry as competitive with whole-cranium deep learning in raw accuracy, but as a parsimonious, interpretable, and reproducible complement that remains applicable when only the sinus region is preserved. Integrating sinus morphometry with deep learning and three-dimensional shape analysis is a promising future direction.

This study has several limitations. It is derived from a single-center Turkish cohort and may not generalize to populations with different mean sinus volumes [28]. Population specificity is a recognized concern in forensic anthropology, because both absolute sinus volumes and the accuracy of sex-prediction models vary among ethnic and geographic groups [25,28]; sinus dimensions reported for South Asian, East Asian, and European populations differ from one another, and a model trained in one population can lose accuracy when applied to another [25]. The volumes and the moderate discrimination observed here should therefore be regarded as population-specific estimates for a Turkish adult sample, and the model requires recalibration and external validation before it is applied to other groups. The sample comprised patients undergoing head CT for clinical indications rather than a healthy reference population, which may introduce selection effects. Although the scans were acquired for indications unrelated to the paranasal sinuses (such as headache, head trauma without facial fracture, or vertigo) and individuals with sinus pathology were excluded, a hospital-based imaging sample is not a random sample of the general population, and the resulting cohort may not be fully representative of the underlying Turkish adult population. The model has not been evaluated in scans with paranasal pathology, post-traumatic deformity, or partial skeletonization—conditions common in forensic casework—and the imaging protocol was fixed, so the influence of different scanners, reconstruction kernels, and slice thicknesses has not been characterized. Finally, no external validation was performed. The model was evaluated only internally, through cross-validation and a single held-out partition drawn from the same cohort; for a forensic prediction model, external validation in independent samples is essential before any practical application, and our findings should be regarded as a model-development result awaiting such validation rather than a deployment-ready tool. A further limitation is that the present analysis was restricted to sinus volume. Contemporary forensic imaging increasingly incorporates additional descriptors—surface area, three-dimensional shape descriptors, geometric-morphometric landmarks, curvature, and texture analysis—which may capture sexually dimorphic information not reflected in volume alone and could improve discrimination beyond the moderate performance reported here.

Future work should prioritize external validation in independent cohorts, ideally from multiple ethnic backgrounds and imaging protocols. Multi-center validation, prospective evaluation in forensic casework, characterization of model behavior in pathological scans, pre-registration of analysis plans, and sharing of de-identified volumetric data [29] would further strengthen the field. More specifically, we recommend that future studies pursue external, multicenter validation across scanners and populations; extend the approach to cone-beam CT (CBCT), which is increasingly used in maxillofacial imaging; incorporate shape-based and geometric-morphometric analyses (landmark configurations, surface area, and curvature) in addition to volume; explore deep learning-based automated segmentation to reduce manual effort and observer dependence; and ultimately compare and, where appropriate, combine sinus morphometry with whole-cranium deep learning models within a single forensic identification pipeline.

## 5. Conclusions

Paranasal sinus volumes in this Turkish adult cohort show robust sex dimorphism, with the frontal sinus as the most discriminative single feature. A cross-validated L1-regularized logistic regression classifier achieves moderate sex-discrimination performance (10-fold cross-validated AUC = 0.79; held-out accuracy 70%), but adds little beyond frontal volume alone; it is therefore appropriate as one corroborating input in a forensic identification workflow rather than as a stand-alone determinant. Age cannot be reliably estimated from sinus morphometry in this cohort. Trained model coefficients are reported transparently to permit external replication.

## Figures and Tables

**Table 1:** Demographic and morphometric characteristics of the study population (*n* = 499). IQR = interquartile range.

Parameter	Value
Age (years), median (IQR)	33 (26–44)
Age range, years	18–65
Sex, *n* (%)	
Male	282 (56.5)
Female	217 (43.5)
Age group, *n* (%)	
18–30 years	202 (40.5)
31–45 years	186 (37.3)
46–65 years	111 (22.2)
Pneumatization pattern, *n* (%)	
Type D (complete sellar)	292 (58.5)
Type C (incomplete sellar)	168 (33.7)
Type B (presellar)	36 (7.2)
Type A (conchal)	3 (0.6)
Asymmetry, *n* (%)	
Left-dominant	201 (40.3)
Right-dominant	183 (36.7)
Symmetric	115 (23.0)
Maxillary volume (cm^3^), median (IQR)	30.7 (25.2–37.2)
Frontal volume (cm^3^), median (IQR)	7.1 (4.7–11.8)
Sphenoid volume (cm^3^), median (IQR)	11.1 (8.4–14.1)
Total volume (cm^3^), median (IQR)	50.6 (40.7–61.6)

**Table 2:** Between-sex comparisons of paranasal sinus volumes (cm^3^). *p* values are from the Mann–Whitney U test, Bonferroni-corrected across four primary outcomes. Effect size: Cliff’s delta; P[M > F] = probability of male superiority. IQR = interquartile range.

Variable	Male, Median (IQR)	Female, Median (IQR)	Median Diff.	Cliff’s δ	P[M > F]	Adj. *p*
Maxillary	34.4 (28.5–41.0)	27.3 (22.6–31.7)	+7.0	0.45	0.73	<0.001
Frontal	10.3 (6.7–14.3)	6.0 (4.0–7.6)	+4.4	0.53	0.77	<0.001
Sphenoid	12.8 (9.4–15.6)	10.5 (7.5–12.8)	+2.6	0.33	0.66	<0.001
Total	57.8 (47.4–67.7)	43.7 (36.2–51.0)	+14.0	0.56	0.78	<0.001

**Table 3:** Performance of the cross-validated sex-prediction model (L1-regularized logistic regression). Ten-fold cross-validation values are reported on the full dataset (*n* = 499); test-set metrics are from the held-out 20% partition (*n* = 100). AUC = area under the receiver operating characteristic curve.

Metric	10-Fold CV (*n* = 499)	Held-Out Test (*n* = 100)
AUC	0.786 ± 0.071	0.796
Accuracy	—	0.700
Sensitivity	—	0.737
Specificity	—	0.651
Precision	—	0.737
F1 score	—	0.737
Brier score	—	0.184

## Data Availability

The full coefficient set of the final model is provided in Supplementary Table S1. De-identified volumetric data are available from the corresponding author upon reasonable request, subject to institutional data-sharing policy.

## Supplementary Materials

The following supporting information can be downloaded at: https://www.mdpi.com/article/10.3390/diagnostics16121928/s1. Table S1: Full coefficient set of the final L1-regularized logistic-regression model for sex estimation, refit on all 499 cases. The outcome is coded as male = 1, female = 0. The three sinus-volume features were z-standardized before fitting; their coefficients therefore apply to standardized values, and the standardization parameters are given in Table S1b. Pneumatization pattern and asymmetry were entered as one-hot (0/1) indicators. Coefficients of exactly 0.000 were removed by the L1 (lasso) penalty. The selected regularization strength was C = 1, chosen by 10-fold stratified cross-validated ROC-AUC on the training data; the final model achieved a 10-fold cross-validated AUC of 0.79. The supplementary file containing the full set of regression coefficients (intercept and per-feature weights on the standardized scale, together with the standardization means and standard deviations required to apply the model) is provided with this revised submission to permit independent replication.
