# Supplementary material for: Paranasal Sinus Morphometry for Forensic Sex Estimation: A Computed Tomography Study of 499 Individuals with a Cross-Validated, Transparently Reported Machine Learning Model
Source: Diagnostics (Basel). 2026 Jun 22;16(12):1928. doi: 10.3390/diagnostics16121928 (PMC13298097; doi:10.3390/diagnostics16121928)
Supplement: Supplementary file 1 [file diagnostics-16-01928-s001.zip › diagnostics-4377221-supplementary.pdf]

Supplementary Table S1.

Full coefficient set of the final L1-regularized logistic-regression model for sex estimation, refit on all 499 cases. The outcome is coded as male = 1, female = 0. The three sinus-volume features were z-standardized before fitting; their coefficients therefore apply to standardized values, and the standardization parameters are given in Table S1b. Pneumatization pattern and asymmetry were entered as one-hot (0/1) indicators. Coefficients of exactly 0.000 were removed by the L1 (lasso) penalty. The selected regularization strength was C = 1, chosen by 10-fold stratified cross-validated ROC-AUC on the training data; the final model achieved a 10-fold cross-validated AUC of 0.79.

Table S1a. Model coefficients (log-odds scale).

Term	Coefficient	Unit / scale
**Intercept**	**0.6566**	—
Maxillary volume (standardized)	0.6293	cm^3
Frontal volume (standardized)	1.0146	cm^3
Sphenoid volume (standardized)	0.4371	cm^3
Pneumatization Type A (vs. others)	0.0000	0/1
Pneumatization Type B (vs. others)	0.0000	0/1
Pneumatization Type C (vs. others)	0.0261	0/1
Pneumatization Type D (vs. others)	−0.6705	0/1
Asymmetry: left-dominant	0.3185	0/1
Asymmetry: symmetric (none)	0.3121	0/1
Asymmetry: right-dominant	0.0000	0/1

Table S1b. Standardization parameters for the numerical features (computed on all 499 cases).

Feature	Mean (cm^3)	SD (cm^3)
Maxillary volume	31.768	10.125
Frontal volume	9.122	6.724
Sphenoid volume	11.539	4.672

Applying the model

For an individual, first standardize each sinus volume as z = (volume − mean) / SD using Table S1b. Then compute the linear predictor:

$$logit(P_{male}) = 0.6566 + 0.6293 \cdot z_{max} + 1.0146 \cdot z_{frontal} + 0.4371 \cdot z_{sphenoid} + c_{pneumatization} + c_{asymmetry}$$

where $c_{pneumatization}$ and $c_{asymmetry}$ are the coefficients from Table S1a for the individual's observed pneumatization pattern and asymmetry category (all other categories contribute 0). The predicted probability of male sex is $P_{male} = 1 / (1 + e^{-logit})$. An individual is classified as male when $P_{male} \geq 0.50$.

Note. *Coefficients were obtained with scikit-learn's L1-penalized LogisticRegression (liblinear solver, C = 1). Because of the L1 penalty, the categorical reference levels and weakly informative indicators (Pneumatization Types*

A and B, and right-dominant asymmetry) were shrunk to exactly zero. The model is intended as one corroborating input in a forensic identification workflow and is population-specific to the Turkish adult cohort studied here.
